# Serum HOTTIP expression is upregulated in nasopharyngeal carcinoma patients and predicts poor prognosis

**DOI:** 10.1016/j.bjorl.2024.101471

**Published:** 2024-07-20

**Authors:** Ding-Ting Wang, Jian Luo, Hua-Jun Feng, Yuan-Yuan Wang

**Affiliations:** a The Affiliated Hospital of Southwest Medical University, Department of Otolaryngology Head and Neck Surgery, Luzhou, China; b The First People’s Hospital of Yibin, Department of Otolaryngology Head and Neck Surgery, Yibin, China

**Keywords:** HOTTIP, Nasopharyngeal carcinoma, Prognosis, Serum

## Abstract

**Objective:**

Nasopharyngeal Carcinoma (NPC) is a tumor with ethnic and geographic distribution characteristics. HOXA Transcript at the distal Tip (HOTTIP) has been confirmed to have cancer-promoting effects in various tumors. The aim of this study was to investigate the clinical significance of HOTTIP in the development of NPC and its role in prognostic assessment.

**Methods:**

Reverse transcription real-time PCR was used to analyze the expression of HOTTIP in the serum of 122 NPC patients before treatment, 35 controls and 30 NPC patients after treatment, and the relationship between HOTTIP expression and the clinicopathological features and prognosis of NPC patients was analyzed. The NPC cell lines CNE1 and HNE1 stably overexpressing HOTTIP were constructed using the retroviral method. Plate clone formation assays, MTT assays, flow cytometry, and Transwell migration and invasion assays were used to investigate the effects of HOTTIP overexpression on the colony-formation ability, cell proliferation ability, apoptosis, and migration and invasion abilities of CNE1 and HNE1 cells, respectively.

**Results:**

HOTTIP was highly expressed in NPC serum and cells compared with the control group; serum HOTTIP expression in NPC patients after treatment was significantly lower than that before treatment; Kaplan-Meier survival curve revealed that the Progression-Free Survival (PFS) and Overall Survival (OS) of the HOTTIP low-expression group were both better than those of the HOTTIP high-expression group; COX proportional hazard models showed that high HOTTIP expression was an independent risk factor for PFS in NPC patients. The overexpression of HOTTIP promoted the proliferation, migration and invasion abilities of CNE1 and HNE1 NPC cells and inhibited cell apoptosis in vitro cell experiments.

**Conclusion:**

Increased HOTTIP expression in serum indicates a poor prognosis and may be used as a molecular marker and therapeutic target in NPC.

**Level of Evidence:**

4.

## Highlights

• HOTTIP was highly expressed in the serum of Nasopharyngeal Carcinoma (NPC).
• HOTTIP expression were related to the PFS and OS of NPC patients.
• High HOTTIP expression was an independent risk factor for PFS in NPC patients
• HOTTIP promoted the proliferation of NPC cells and inhibited cells apoptosis.

## Introduction

Nasopharyngeal Carcinoma (NPC) is a malignant tumor prone to occur in the roof of the nasopharynx and mucosal epithelium of pharyngeal crypts, and southern China accounts for a large number of cases.^1^ In 2020, there were 133,354 new cases of NPC in 185 countries around the world, with approximately 80,000 deaths.^2^ With advancements in intensity-modulated radiotherapy and concurrent chemoradiotherapy, the survival rate for patients with early NPC has improved.^3^ However, NPC is poorly differentiated, metastasizes early, and recurs easily; therefore, the vast majority of patients have local lymph node and/or distant metastasis at the time of treatment, and their prognosis is relatively poor.^4^ Studies have shown that the pathogenesis of NPC is closely related to factors such as Epstein–Barr Virus (EBV) infection, genetics, environment, lifestyle, and diet^5^; however, the molecular mechanisms of the pathogenesis of NPC have not been fully elucidated to date.

Long non-coding RNAs (lncRNAs) are a type of RNA with transcripts more than 200 nucleotides in length that are involved in multiple molecular regulatory processes, such as genomic imprinting, X chromosome silencing, transcription interference, and chromatin modification, and are inseparable from the normal physiological function of the human body and the occurrence and development of some diseases.^6^, ^7^, ^8^, ^9^, ^10^ HOXA Transcript at the distal Tip (HOTTIP) is located on human chromosome 7q15.2 and is transcribed from the antisense strand at the 5' end of the HOXA gene cluster. It mainly binds to the WDR5/MLL complex to promote the methylation of H3K4 and thus the transcriptional activation of the terminal gene HOXA to promote the expression of development-related genes.^11^ Our research team found that HOTTIP expression was increased in NPC tissues and cell lines and that HOTTIP silencing inhibited the proliferation, migration and invasion of NPC cells.^12^ However, the expression of HOTTIP in the serum of NPC patients and the effect HOTTIP overexpression on the biological properties of NPC cells have not been reported.

With the advent of “liquid biopsies”, such as circulating tumor DNA, extracellular vesicles, and circulating RNA, the detection of tumor components in blood and other body fluids has become a new method for cancer diagnosis, accurate treatment and prognosis prediction.^13^ EBV antibody and EBV-DNA are commonly used clinical NPC screening items, which positivity rate and DNA copy number have definite correlations with the TNM stage and prognosis of patients with NPC, but the specificity and sensitivity need to be improved.^14^, ^15^, ^16^ In this study, the expression levels of HOTTIP in the serum of NPC patients were analyzed to investigate the role of HOTTIP in the clinicopathological features and prognosis evaluation of NPC patients. HOTTIP expression in NPC cells was upregulated to further study the effect of HOTTIP overexpression on the biological characteristics of NPC cells.

## Methods

### Specimens

A total of 122 NPC patients hospitalized in the Department of Otorhinolaryngology Head and Neck Surgery at the Affiliated Hospital of Southwest Medical University between August 2016 and November 2018 and 35 healthy control subjects who did not have tumor diagnoses or other major disease diagnoses were enrolled. The inclusion criteria of the case group were: newly diagnosed primary NPC patients confirmed by clinicopathology; did not receive radiotherapy, chemical drugs and other treatments before biopsy and blood drawing; no personal history of tumor and recent history of blood transfusion. Blood was collected immediately after patient was diagnosed with NPC, before them had started treatment. Venous blood was collected in blood collection tubes without anticoagulant. Serum was obtained by centrifugation at 3,000 rpm for 10 min and stored in a −80 °C freezer. The whole process was completed within 4 h after blood collection. NPC was confirmed by a pathological examination. TNM staging was performed using the TNM staging criteria of the American Cancer Society (7th edition, 2010). All samples were collected with participants' consent and signed informed consent. This study was approved by the Ethics Committee of the Affiliated Hospital of Southwest Medical University.

### Cell culture and establishment of stable HOTTIP-overexpressing cell lines

CNE1 and HNE1 NPC cells were cultured in RPMI-1640 medium (Gibco, USA) containing 10% FBS + 1% penicillin and streptomycin. The NP69 immortalized nasopharyngeal epithelial cell line (Gibco, USA) was cultured in keratinocyte-SFM medium (Gibco, USA). The cells were cultured at 37 °C in 5% CO_2_ in a saturated humidity incubator. CNE1 and HNE1 cells in logarithmic growth phase were seeded in 6-well plates. A constructed vector, i.e., pBABEpuro, was transfected into platA retroviral packaging cells using transfection reagent. The viral supernatant was collected after 48 h, and 1.5 mL of fresh cell culture medium and 2 μL of polybrene were added to 0.5 mL of viral supernatant. The premixed viral infection solution was added to the cell culture dishes, and then, the culture was continued for 2 days. Then, CNE1 and HNE1 cells that stably overexpressed HOTTIP (CNE1-HOTTIP, HNE1-HOTTIP) were selected using puromycin (InvivoGen, USA). Additionally, CNE1 and HNE1 cells stably infected with empty vectors (CNE1-NC and HNE1-NC) were prepared.

### RNA extraction and reverse transcription real-time fluorescence quantitative PCR (RT‒qPCR)

Total RNA was extracted using RNAiso Plus (TaKaRa, Japan) following the manufacturer’s instructions. The concentration and purity of RNA were assessed with an analyzer. RNA was converted to cDNA by reverse transcription following the manufacturer’s instructions. cDNA was used as a template for real-time fluorescence quantitative PCR. The amplification reaction was performed using 2 × SYBR Green master mix (Invitrogen, USA). The relative amount of HOTTIP was measured in an ABI7500 fluorescence PCR instrument (ABI, USA), and GAPDH was used as an internal reference. The primer sequences for HOTTIP were as follows: HOTTIP-F: CAGCTCTCAGGGAAACGAAG and HOTTIP-R: CCTCTCCAACCCATAACTG. All experiments were repeated three times. The relative expression level of HOTTIP in each cell line was calculated by the 2^−ΔΔCt^ method (ΔCt = Ct _HOTTIP_-Ct _GAPDH_). Similarly, the relative expression level of HOTTIP in serum was measured using β-actin as an internal reference. The HOTTIP primer sequences were as follows: HOTTIP-F: AAGGCACTCACTTCTTC and HOTTIP-R: ACAGGCAGGGCTGTAACTCAA.

### Plate colony formation assay

CNE1 and HNE1 cells with good growth status were collected, the cells were diluted to 1,000 cells/mL, and 1.5 mL of complete medium and 200 μL of diluted cell suspension were added to each well of a 6-well culture plate. After culturing for 1–2 weeks, when cell colonies visible to the naked eye appeared, the cells were fixed with 4% paraformaldehyde for 30 min, stained with crystal violet for 30 min, washed with staining solution, and dried at room temperature. The number of clones was counted under an inverted microscope (one cell cluster ≥50 cells was counted as 1 colony). Colony formation rate = number of clones per well/number of seeded cells per well ×100%.

### MTT assay

CNE1 and HNE1 cells with good growth status were collected, diluted to 5,000 cells/mL, and seeded in 96-well plates with 200 μL of cell suspension per well. Each group had four replicate wells. A blank control with medium only was also set up. After 24 h, 48 h, 72 h and 96 h of culture, 20 µL of MTT solution was added to each well in the dark. After 4 h, the culture was terminated. The blank control well was used to set zero, and the Optical Density (OD) value of each well was measured at 490 nm in a microplate reader.

### Cell apoptosis analyzed by flow cytometry

CNE1 and HNE1 cells with good growth status were collected, washed twice with PBS, washed once with 1× binding buffer, and resuspended in 1× binding buffer to a cell concentration to 1.0 × 10^6^ cells/mL; then, 5 μL of FITC Annexin V and 5 μL of Propidium Iodide (PI) were added to the cell suspension, which was mixed evenly and incubated in the dark for 10 min, followed by flow cytometry.

### Transwell migration and invasion assays

CNE1 and HNE1 cells with good growth status were collected, and the cell concentration in each group was adjusted to 5 × 10^5^ cells/mL in serum-free RPMI-1640 medium. A total of 200 μL of the above cell suspension was added to the upper chamber of the Transwell (Corning, USA), and 600 μL of RPMI-1640 medium containing 10% FBS was added to the lower chamber. The cells were incubated for 24 h, fixed with 4% paraformaldehyde for 30 min, stained with crystal violet for 30 min, and examined under a microscope. Four fields for each small chamber were photographed and counted. For the invasion experiment, 100 μL of diluted Matrigel (BD, USA) was evenly spread on the Transwell membrane, and the other steps were the same as those in the migration experiment.

### Follow-up

A total of 122 newly diagnosed NPC patients were followed up. The follow-up contents included a blood draw after obtaining informed consent; recurrence and metastasis in and the survival status of the registered patients were recorded. The last follow up date was January 31, 2019. The time after treatment was calculated starting with the end of all radiotherapy and chemotherapy. Among the 122 NPC patients, 30 NPC patients provided whole blood 1 month after treatment for serum isolation.

### Statistical analysis

All data were analyzed using SPSS 22.0 software. Measurement data are expressed as the Mean ± SD. Comparisons between two groups were performed using the *t*-test, and comparisons among multiple groups were performed using one-way analysis of variance (ANOVA). The relationship between the expression level of HOTTIP and the prognosis of NPC patients was determined using the *χ*^2^ test. Survival analysis was performed using the Kaplan–Meier (K–M) method with the log-rank test. A Cox proportional hazards model was used to analyze the prognostic factors; *p* < 0.05 was considered statistically significant.

## Results

### Expression of HOTTIP in NPC serum

**Fig. 1:**
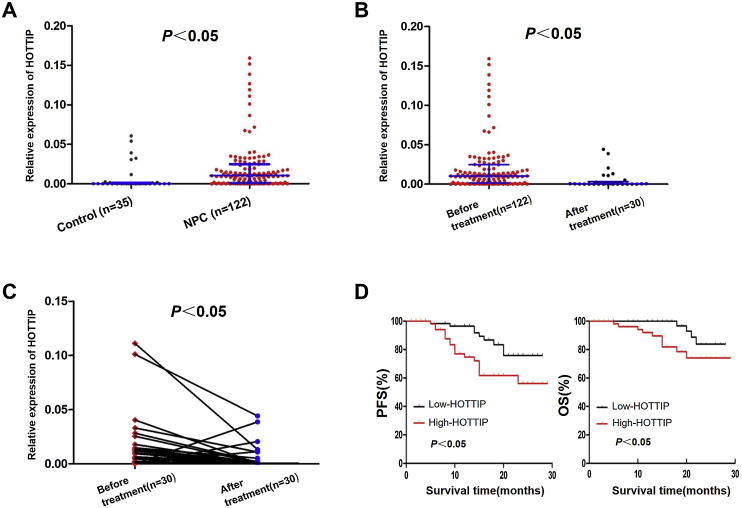
Expression of HOTTIP in serum of patients with nasopharyngeal carcinoma and its relationship with prognosis. (A) HOTTIP is highly expressed in NPC serum. (B) HOTTIP expression in 30 NPC patients after treatment was significantly lower than that in 122 NPC patients before treatment. (C) HOTTIP expression in 30 NPC patients after treatment decreased significantly compared with that before treatment. (D) Kaplan-Meier curves of PFS and OS in NPC patients with different HOTTIP expression levels. OS, Overall Survival; PFS, Progression-Free Survival.

The expression of HOTTIP in the serum was significantly higher in NPC patients than in the control group (*p* < 0.05, Fig. 1A). There was no significant difference in HOTTIP expression between groups by age, sex, pathological type, T-stage, N-stage, M-stage, or clinical stage (*p* > 0.05, Table 1). The relative expression level of HOTTIP in the serum of 122 NPC patients before treatment and 30 NPC patients after treatment was compared, and the results showed that HOTTIP expression in NPC patients after treatment was significantly lower than that before treatment (*p* <  0.05, Fig. 1B). A comparison of the relative expression level of HOTTIP in 30 NPC patients who provided pretreatment and posttreatment serum specimens showed that after treatment, HOTTIP expression decreased in 25 patients and increased in 5 patients (Table 2). HOTTIP expression was significantly lower after treatment than before treatment (*p* < 0.05, Fig. 1C).

**Table 1:** Clinicopathological features of NPC patients and their relationship with serum HOTTIP expression.

Features	Number of cases	HOTTIP expression	p-value
Gender			
Male	88	0.025 ± 0.040	0.192
Female	34	0.015 ± 0.023	
Age(years)			
≥52	70	0.024 ± 0.043	0.412
<52	52	0.019 ± 0.026	
Pathologic type^a^			
I	4	0.013 ± 0.016	
II	34	0.026 ± 0.044	0.789
III	84	0.021 ± 0.034	
T stage			
T1	18	0.032 ± 0.056	0.195
T2	33	0.018 ± 0.026	
T3	44	0.019 ± 0.027	
T4	27	0.030 ± 0.042	
N stage			
N0	10	0.009 ± 0.011	0.159
N1	29	0.017 ± 0.025	
N2	65	0.029 ± 0.045	
N3	18	0.013 ± 0.011	
M stage			
M0	114	0.021 ± 0.036	0.247
M1	8	0.036 ± 0.038	
Clinical stage^b^			
Early	15	0.007 ± 0.009	0.107
Advanced	107	0.024 ± 0.038	

a I: Keratinizing squamous cell carcinoma; II: Non-keratinized differentiated carcinoma; III: Non-keratinized undifferentiated carcinoma.

b Early: TNM stage I–II; Advanced: TNM stage III–IV.

**Table 2:** Changes of serum HOTTIP expression before and after treatment in 30 NPC patients.

Patient number	Expression of HOTTIP before treatment	Expression of HOTTIP after treatment
1^a^	0.00083	0.01136
13	0.01795	0.00010
31	0.01243	0.00119
35	0.00648	0.00185
47	0.00090	0.00002
49	0.01031	0.00017
53	0.00230	0.00012
57	0.01201	0.00054
61	0.10111	0.04419
63^a^	0.00000	0.02062
65	0.11111	0.01323
67^a^	0.00032	0.00258
105	0.01468	0.00523
117	0.00193	0.00054
147	0.00025	0.00016
159	0.02538	0.00083
161	0.01305	0.00002
169	0.00152	0.00015
183^a^	0.00013	0.03874
187	0.01795	0.00071
191	0.02836	0.00160
193	0.01458	0.00003
211	0.00545	0.00005
225	0.04039	0.00049
239	0.00078	0.00005
247	0.00458	0.00001
271	0.00617	0.00016
283	0.03303	0.01090
293	0.00936	0.00049
295^a^	0.00002	0.00005

a Patient with elevated HOTTIP expression after treatment.

### Relationship between HOTTIP expression level and the prognosis of NPC patients

Up to the end of follow-up, 27 of 122 patients had recurrence or metastasis, and the overall Progression-Free Survival (PFS) rate was 77.87% (95/122); 14 patients died, all of whom died from the disease, and the Overall Survival (OS) rate was 88.52% (108/122). Using the median relative expression level of HOTTIP (0.01045), 122 NPC patients were divided into high-expression (n = 62) and low-expression (n = 60) groups. The statistical analysis revealed that in the high expression group of HOTTIP, the PFS rate was 70.97% (44/62) and the OS rate was 83.87% (52/62); whereas in the low expression group of HOTTIP, the PFS rate reached 85.00% (51/60) and the OS rate achieved 93.33% (56/60). K–M survival curves showed significantly better PFS and OS in the HOTTIP low-expression group than in the HOTTIP high-expression group (*p* < 0.05, Fig. 1D). A Cox proportional hazards model was used to analyze the effect of serum HOTTIP expression level and other clinicopathological factors (clinical stage, T-stage, N-stage, etc.) on the prognosis of patients. The results showed that the HOTTIP expression level and M-stage were independent risk factors for PFS in NPC patients (*p* < 0.05, Table 3).

**Table 3:** Cox regression analysis of PFS and OS in NPC patients.

Variables	PFS		OS	
	HR (95% CI)	p-value	HR (95% CI)	p-value
Gender: male vs. female	0.620 (0.236, 1.632)	0.333	0.819 (0.220, 3.051)	0.766
Age: ≥52 vs. <52	1.925 (0.648, 1.351)	0.147	3.199 (0.819, 12.497)	0.094
Pathologic type^a^: Ⅰ, Ⅱ, Ⅲ	0.940 (0.410, 2.157)	0.884	0.729 (0.248, 2.140)	0.585
Clinical stage^b^: early vs. advanced	0.582 (0.201, 1.690)	0.320	0.476 (0.097, 2.344)	0.361
T stage: T1, T2, T3, T4	1.759 (0.897, 3.451)	0.100	2.059 (0.779, 5.438)	0.145
N stage: N0, N1, N2, N3	1.221 (0.603, 2.473)	0.580	1.396 (0.521, 3.741)	0.508
M stage:M0 vs. M1	4.826 (1.556, 14.967)	0.006	5.636 (1.285, 24.721)	0.022
HOTTIP level: low vs. high	2.568 (1.048, 6.295)	0.039	4.000 (0.950, 16.849)	0.059

OS, Overall Survival; PFS, Progression-Free Survival.

a I: Keratinizing squamous cell carcinoma; II: Non-keratinized differentiated carcinoma; III: Non-keratinized undifferentiated carcinoma.

b Early: TNM stage I–II; Advanced: TNM stage III–IV.

### Expression of HOTTIP in NPC cells

**Fig. 2:**
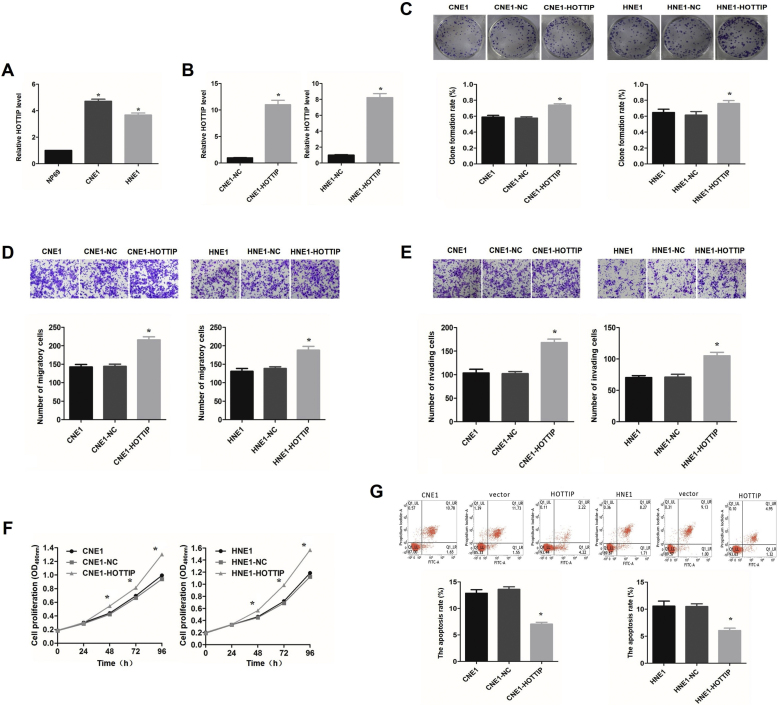
The effect of overexpression of HOTTIP on NPC cells. (A) HOTTIP is highly expressed in CNE1 and HNE1 cells. (B) The expression level of HOTTIP in CNE1 and HNE1 cells after transfection. (C) The effects of overexpression of HOTTIP on the colony-forming ability of CNE1 and HNE1 cells. (D) The effects of overexpression of HOTTIP on the migration ability of CNE1 and HNE1 cells. (E) The effects of overexpression of HOTTIP on the invasion ability of CNE1 and HNE1 cells. (F) The effects of overexpression of HOTTIP on the proliferation ability of CNE1 and HNE1 cells. (G) The effects of overexpression of HOTTIP on the apoptosis of CNE1 and HNE1 cells. *p < 0.05, compared with control group.

The expression of HOTTIP was significantly higher in the CNE1 and HNE1 NPC cell lines than in the NP69 immortalized nasopharyngeal epithelial cell line (*p* < 0.05, Fig. 2A). To verify the establishment stable HOTTIP-overexpressing CNE1 and HNE1 cell lines, the expression of HOTTIP in each group was analyzed by RT-qPCR. The results showed that HOTTIP expression was significantly higher in the overexpression group than in the empty vector group (*p* < 0.05, Fig. 2B).

### HOTTIP overexpression enhanced the proliferation, invasion and metastasis abilities of NPC cells

The results of the plate colony formation assay showed that the colony formation rates of CNE1 and HNE1 cells were significantly higher in the overexpression group than in the normal cell group (CNE1, HNE1) and empty vector group (*p* < 0.05, Fig. 2C). Transwell migration assays showed that the number of CNE1 and HNE1 cells that migrated was significantly higher for the HOTTIP-overexpressing cells than for the normal cells (CNE1 and HNE1) and cells in the empty vector group (*p* < 0.05, Fig. 2D). Transwell invasion assays showed that the number of CNE1 and HNE1 cells that migrated crossed was significantly higher for the overexpression group than for the normal cells (CNE1 and HNE1) and cells in the empty vector group (*p* < 0.05, Fig. 2E). MTT assays were performed to assess the proliferation ability of cells in each group (0 h, 24 h, 48 h, 72 h, and 96 h). The results showed that starting at 48 h, the proliferation ability of the HOTTIP-overexpressing CNE1 and HNE1 cells was significantly stronger than that of the normal cells (CNE1 and HNE1) and the cells in the empty vector group, with the proliferation ability gradually increasing over time (*p* < 0.05, Fig. 2F).

### HOTTIP overexpression inhibited NPC cell apoptosis

Flow cytometry revealed that the apoptosis rates of CNE1 and HNE1 cells were significantly lower in the HOTTIP-overexpressing group than in the normal cells (CNE1 and HNE1) and cell in the empty vector group (*p* < 0.05, Fig. 2G).

## Discussion

Studies have found that lncRNAs, such as HOTAIR, HOXA9, MALAT1, and FOXP4-AS1, play important regulatory roles in NPC proliferation, apoptosis, invasion and metastasis^17^, ^18^, ^19^, ^20^, ^21^ and that they are mainly located in tissues; however these lncRNAs can also exist in various body fluids in a free state, in a bound state or in exosomes.^19^, ^20^ HOTTIP is abnormally expressed in the serum of patients with gastric cancer, colorectal cancer, liver cancer, and non-small cell lung cancer, and the increase or decrease in HOTTIP expression can be used as a potential diagnostic and prognostic molecular marker.^22^, ^23^, ^24^, ^25^ To explore the relationship between serum HOTTIP and NPC, in this study, the expression of HOTTIP in the serum of NPC patients was analyzed first. The results showed that the expression level of HOTTIP was significantly higher in NPC patients than in the control group, suggesting that HOTTIP plays an important role in the development of NPC. The detection of serum HOTTIP expression may be used for the early screening of NPC.

Prognosis is the ultimate evaluation standard of tumor diagnosis and treatment. In this study, the expression level of HOTTIP in NPC patients was significantly lower after treatment than before treatment. These results suggest that chemotherapy and radiotherapy for NPC can effectively reduce the expression level of HOTTIP in patients. Our research team speculated that this might be related to the reduced tumor burden in NPC patients and thus that monitoring serum HOTTIP levels is helpful for evaluating the treatment effect of NPC patients. More importantly, in the present study, the PFS and OS were better in the HOTTIP low-expression group than in the HOTTIP high-expression group. High HOTTIP expression is an independent risk factor for NPC, indicating that the detection of serum HOTTIP expression levels has the potential to predict the prognosis of NPC patients. High HOTTIP expression may suggest a poor prognosis for NPC patients.

Studies have shown that HOTTIP silencing can promote miR-4301 expression and thus inhibit NPC cell proliferation, migration and invasion.^26^ A previous study by our research team found that HOTTIP promoted tumorigenesis by regulating HOXA13 expression in NPC cells.^12^ Further studies showed that HOXA13 promotes the proliferation, migration and invasion of NPC HNE1 cells by upregulating Snail and MMP-2.^27^ This shows the diversity and uncertainty of HOTTIP as an oncogene in the pathogenesis and development of NPC. In this study, by upregulating HOTTIP expression in NPC cells, it was found that the proliferation, invasion and metastasis of NPC cells were enhanced, and apoptosis was decreased. On the basis of previous studies on HOTTIP expression inhibiting,^12^ it is further suggested that HOTTIP plays a role as an oncogene in NPC, laying an experimental foundation for the search for NPC related target genes and signaling pathways.

As we all know, the occurrence and development of NPC is a complex process, and the prognosis of patients is affected by many factors, of which HOTTIP is only one factor. The limitation of this study is that the sample size is small and the changes of serum HOTTIP in NPC patients cannot be dynamically detected at multiple time nodes for some reasons. In the future, it is necessary to continuously optimize the research process and strive to collect clinical samples at multiple time points after treatment, which will be an interesting and meaningful study to evaluate the application value of HOTTIP as a molecular marker for NPC prognosis.

## Conclusions

In summary, the present study revealed for the first time that HOTTIP was increased in the serum of NPC patients and that its expression decreased after treatment. Increased HOTTIP expression in serum indicates a poor prognosis and may be used as a molecular marker and therapeutic target in NPC.

## Funding

This work was supported by grants from the Southwest Medical University Fund Project (grant number 2019-ZRQN-123).

## Conflicts of interest

The authors declare no have conflicts of interest.
